# RAS mutation associated with short surgically controllable period in colorectal liver metastases: a retrospective study

**DOI:** 10.1186/s12957-024-03529-9

**Published:** 2024-09-12

**Authors:** Sono Ito, Takeshi Takamoto, Satoshi Nara, Daisuke Ban, Takahiro Mizui, Hiroshi Nagata, Yasuyuki Takamizawa, Konosuke Moritani, Shunsuke Tsukamoto, Yukihide Kanemitsu, Yusuke Kinugasa, Minoru Esaki

**Affiliations:** 1 Department of Hepatobiliary and Pancreatic Surgery, National Cancer Center Hospital, Tokyo, 104-0045 Japan; 2 Department of Colorectal Surgery, National Cancer Center Hospital, Tokyo, 104-0045 Japan; 3 Department of Gastrointestinal Surgery, Tokyo Medical and Dental University, Tokyo, 113-8519 Japan

**Keywords:** Colorectal cancer, Liver metastasis, RAS, Surgically controllable period, Neoadjuvant chemotherapy

## Abstract

**Background:**

The prognostic implications of the RAS status in colorectal cancer liver metastasis (CRLM) remain unclear. This study investigated the prognostic significance of RAS status after curative hepatectomy, focusing on surgical controllability.

**Methods:**

This retrospective study included liver-only CRLM patients who underwent the first hepatectomy between 2015 and 2022 at the National Cancer Center Hospital. Recurrence-free survival (RFS), surgically controllable period (SCP), and overall survival (OS) were compared between RAS wild-type (RAS-wt) and mutant (RAS-mt) patients. Multivariate analyses were conducted to identify independent prognostic factors for each outcome and independent risk factors for less than 1 year SCP.

**Results:**

A total of 150 patients were evaluated, comprising 63 patients with RAS-mt status. There was no significant difference in RFS between RAS-mt and RAS-wt (7.00 vs. 8.03 months, *p* = 0.48). RAS-mt patients exhibited worse SCP (11.80 vs.21.13 months, *p* < 0.001) and OS (44.03 vs. 70.03 months, *p* < 0.001) compared to RAS-wt. Multivariate analysis identified RAS-mt as an independent prognostic factor for both OS (hazard ratio [HR]: 3.37, *p* < 0.001) and SCP (HR: 2.20, *p* < 0.001), and as an independent risk factor for less than 1 year of SCP (odds ratio, 2.31; *p* = 0.03).

**Conclusions:**

CRLM with RAS mutations should be considered for strict surgical indications with preoperative chemotherapy and thorough examination, considering the possibility of short SCP.

**Supplementary Information:**

The online version contains supplementary material available at 10.1186/s12957-024-03529-9.

## Introduction

Colorectal cancer is the third most common cancer worldwide, with an estimated over 1,926,000 new cases and 903,000 deaths reported in 2022, accounting for 9.6% of new cancer cases and 9.3% of cancer deaths [1, 2]. The liver is the most common metastatic organ of colorectal cancer, with 27% of colorectal cancer patients developing liver metastases during their course [3]. Several systemic chemotherapeutic drugs and regimens for metastatic colorectal cancer have been developed. These treatments yield a progression-free survival of 10–12 months [4, 5] and an objective response rate of up to 65% [5]. Liver resection remains an optimal curative option for liver metastases [6, 7], with repeat hepatectomy recommended to enhance prognosis following initial hepatectomy [8, 9]. Recent trials, such as CAIRO5, have demonstrated promising outcomes, achieving an R0/1 resection rate of 51% for previously unresectable liver metastasis when combined with chemotherapy [10]. This approach holds the potential for achieving long-term survival and possibly curing colorectal cancer liver metastases (CRLMs).

However, not all CRLM patients experience prolonged survival following surgery or chemotherapy, and only a quarter of CRLM patients achieve recurrence-free survival (RFS) of five years or more after hepatectomy [11]. Given the surgical invasiveness and potential for postoperative complications, it is critical to identify patients who would derive meaningful benefit from hepatectomy. Various risk assessment tools have been developed [12–14], with a few recently developed models incorporating RAS status [15, 16]. Nevertheless, the prognostic impact of the RAS mutation status remains controversial. Takeda et al. pointed out that this depends on the characteristics of the patient population, such as the percentage of preoperative chemotherapy administered [17]. RAS mutation has also been reported to cause lung recurrence [18, 19]. The RAS status is an important piece of information when considering treatment strategies for CRLM. Thus, the influence of RAS mutations on overall survival (OS) and RFS has been extensively discussed, yet definitive conclusions remain elusive.

In this study, we aimed to explore the impact of RAS mutations on disease-surgically-controllable intervals post-surgery and its implications for prognosis.

## Materials and methods

### Study population

From our prospectively maintained database, consecutive patients who underwent initial hepatectomy and were histologically diagnosed with CRLMs between January 2015 and September 2022 were identified. Patients with an unknown genomic DNA status of RAS and BRAF were excluded. This retrospective study was approved by the institutional review board of the National Cancer Center Hospital (approval no. NCCH-2018-299).

### Patient treatment

The treatment strategy was determined after a multidisciplinary team (MDT) conference. The primary tumor and liver metastases were resected simultaneously if both were anatomically resectable with synchronous metastasis. Liver resection was conducted only when the primary lesion had been removed or was scheduled for removal and if all the liver metastases were technically resectable. Our institution did not employ a liver-first approach during the study period. Systemic chemotherapy was administered for technically unresectable liver metastases, with conversion surgery performed upon sufficient tumor shrinkage to allow resection. The final treatment strategy was determined after a MDT conference. All patients underwent preoperative volumetry and evaluation of liver functional reserve using the indocyanine green retention test to calculate the minimum required remnant liver volume [20].

After liver resection, patients were regularly followed up by laboratory and imaging evaluations every 3–4 months for five years. Routine adjuvant chemotherapy was not administered. If recurrence was detected within 6 months of liver resection, 3–6 courses of systemic chemotherapy were administered, even if it was technically resectable. The treatment regimen was determined in an MDT conference. If the patient’s response was anything other than progressive disease (PD), they were reassessed in the MDT conference. Patients underwent surgical treatment if R0 or R1 resection was possible. This strategy applied not only to residual liver recurrence but also to extrahepatic metastasis.

### Definitions

In addition to the standard endpoints of OS and RFS after hepatectomy, we evaluated recurrence status in terms of amenability to further surgical treatment. A recurrence status exceeding additional surgical treatments was defined as surgical failure (SF), and the time from the date of hepatectomy to the diagnosis of SF was defined as the surgically controllable period (SCP).

As our definition of SF differs slightly from that of Oba et al. [21], we used the definition of SCP in this study to distinguish it from their “time to SF (TSF).” Patients with resectable recurrences and an RFS of less than 6 months are defined as having early recurrence and receive pseudo-neoadjuvant chemotherapy (p-NAC) at our institute. Undergoing systemic chemotherapy for recurrence does not necessarily indicate SF. SF is defined as the point at which the tumor reaches PD after p-NAC and is deemed unresectable. We perform p-NAC on patients with early recurrence and assess its effectiveness to determine surgical controllability. Consequently, a short SCP is defined as an SCP of less than one year.

OS was defined as the time from the date of hepatectomy to the date of death from any cause or last confirmed alive. RFS was defined as the period from the date of hepatectomy to the date of recurrence or death from any cause.

The following clinicopathological data were collected from the prospective database: age, sex, T and N stage of the primary tumor, timing of metastasis (synchronous or metachronous), number of liver metastases, size of liver metastasis, unilobar or bilobar liver metastases, pre-hepatectomy chemotherapy, post-hepatectomy chemotherapy, resection margin after hepatectomy, carcinoembryonic antigen (CEA) level, carbohydrate antigen 19 − 9 (CA 19-9) level, and RAS gene status. According to the Beppu nomogram, the cut-off values for CEA and CA 19 − 9 levels were set at 100 ng/mL and 100 U/mL, respectively. The cutoffs for the size and number of CRLMs were set at 5 cm and 5, respectively [12]. A margin of less than 1 mm was defined as R1.

Patients with genetic data underwent sequencing of the following RAS codons: KRAS codon 12, 13, 59, 61, 117, 146, NRAS codon 12, 13, 59, 61, 117, 146. All tumors were collected from biopsies or curative surgical resection. Genomic DNA was extracted from formalin-fixed paraffin-embedded tissues after microdissection. The assay was performed using the polymerase chain reaction-reverse sequence-specific oligonucleotide method. Until March 2015, mutation screening was performed for KRAS codons 12 and 13. After April 2015, mutation screening was performed for the KRAS and NRAS codons 12, 13, 59, 61, 117, and 146. Either primary or metastatic tissue was accepted for the measurements, as a high concordance of RAS mutational status between primary and corresponding metastases has been reported [22].

The right-sided colon includes the cecum, ascending colon, and transverse colon, whereas the left-sided colon includes the descending colon, sigmoid colon, and rectum. Tumor markers were used before liver resection.

### Statistics

Clinicopathological characteristics and surgical outcomes were compared between RAS mutant (RAS-mt) and RAS wild-type (RAS-wt) groups. A subanalysis was also performed for KRAS codon 12 and 13 mutations. Continuous variables were summarized as medians [minimum and maximum values]. Categorical variables were expressed as the number of patients and frequencies (percentages). The Mann–Whitney U test was used to compare continuous variables, while Fisher’s exact test was used to compare categorical variables. The Kaplan–Meier method was used to estimate the survival function of OS, SCP, and RFS after hepatectomy. To identify the prognostic factors of OS, SCP, and RFS after hepatectomy, Cox regression analyses were performed, and hazard ratios (HRs) and corresponding 95% confidence intervals (CIs) were estimated for each endpoint. The likelihood of short SCP was expressed as odds ratios (ORs) and 95% CIs. These analyses included all the factors described in the Evaluation Factors section. Univariate analyses were performed to identify potentially relevant factors that were entered into the multivariable analysis if the univariate analyses provided a two-sided p-value of < 0.1. Statistical significance was set at p-value < 0.05. All statistical analyses were performed using the IBM SPSS Statistics 29.

## Results

During the study period, 315 patients underwent an initial hepatectomy for CRLM. Eighteen patients with unresected extrahepatic metastasis were excluded. Among the 297 patients who underwent initial curative hepatectomy, genetic data were available for 154. Four patients with BRAF mutations were excluded from the analysis because they were included in the clinical trials and 150 patients were included. Sixty-three patients were categorized into RAS-mt group, and 87 patients were categorized into RAS-wt group. A right-sided primary tumor, a more invasive primary tumor, higher tumor marker levels, and preoperative chemotherapy induction were more frequently observed in RAS-mt than in RAS-wt. The details are presented in Table 1. In addition, details of preoperative chemotherapy are shown in Supplementary Table 1.

**Table 1:** Patient characteristics by RAS status

Category	value	RAS				
		Wild (*n* = 87)		Mutant (*n* = 63)		P value
Age	Median [min - max]	60.0	[26–82]	63.0	[26–85]	0.54
Sex	Female	29	(33%)	31	(49%)	0.06
Sidedness	Right	10	(11%)	24	(38%)	< 0.001*
	Left	77	(89%)	39	(62%)	
T stage	T1–2	15	(17%)	3	(5%)	0.02*
	T3–4	72	(83%)	60	(95%)	
N stage	N+	52	(60%)	40	(63%)	0.74
Time to CRLM	Synchronous	59	(68%)	39	(62%)	0.49
CRLM number	Median [min - max]	3	[1–24]	3	[1–11]	0.77
CRLM size	Median [min - max]	3.5	[0.3–13.0]	3.0	[0.7–10.4]	0.17
Bilobar liver disease	Bilobar	42	(48%)	35	(56%)	0.41
Prehepatic resection chemotherapy	Present	41	(47%)	16	(25%)	0.01*
Posthepatic resection chemotherapy	Present	14	(16%)	10	(16%)	1
Pathological resection margin	+	23	(26%)	13	(21%)	0.45
CEA (ng/mL)	Median [min - max]	10	[1–2302]	19.5	[0.7–1298]	0.03*
CA 19 − 9 (U/mL)	Median [min - max]	17.5	[1–1204]	60	[1–9720]	< 0.001*

Abbreviations: y/o, years old; CRLM, colorectal cancer liver metastasis; CEA, carcinoembryonic antigen; CA, carbohydrate antigen

*: *p* < 0.05

### Prognosis

Complete follow-up was conducted for the entire cohort of patients, and the median length of follow-up for all included patients was 29.18 [0.57–81.93] months. The median OS was 58.87 [53.23–64.50] months, and the 5-year OS rate was 47.5%. The median SCP was 15.07 [11.23–18.90] months, and the 5-year SCP rate was 24.1%. The median RFS was 7.73 [6.09–9.38] months and the 5-year RFS rate was 5.5%.

**Fig. 1:**
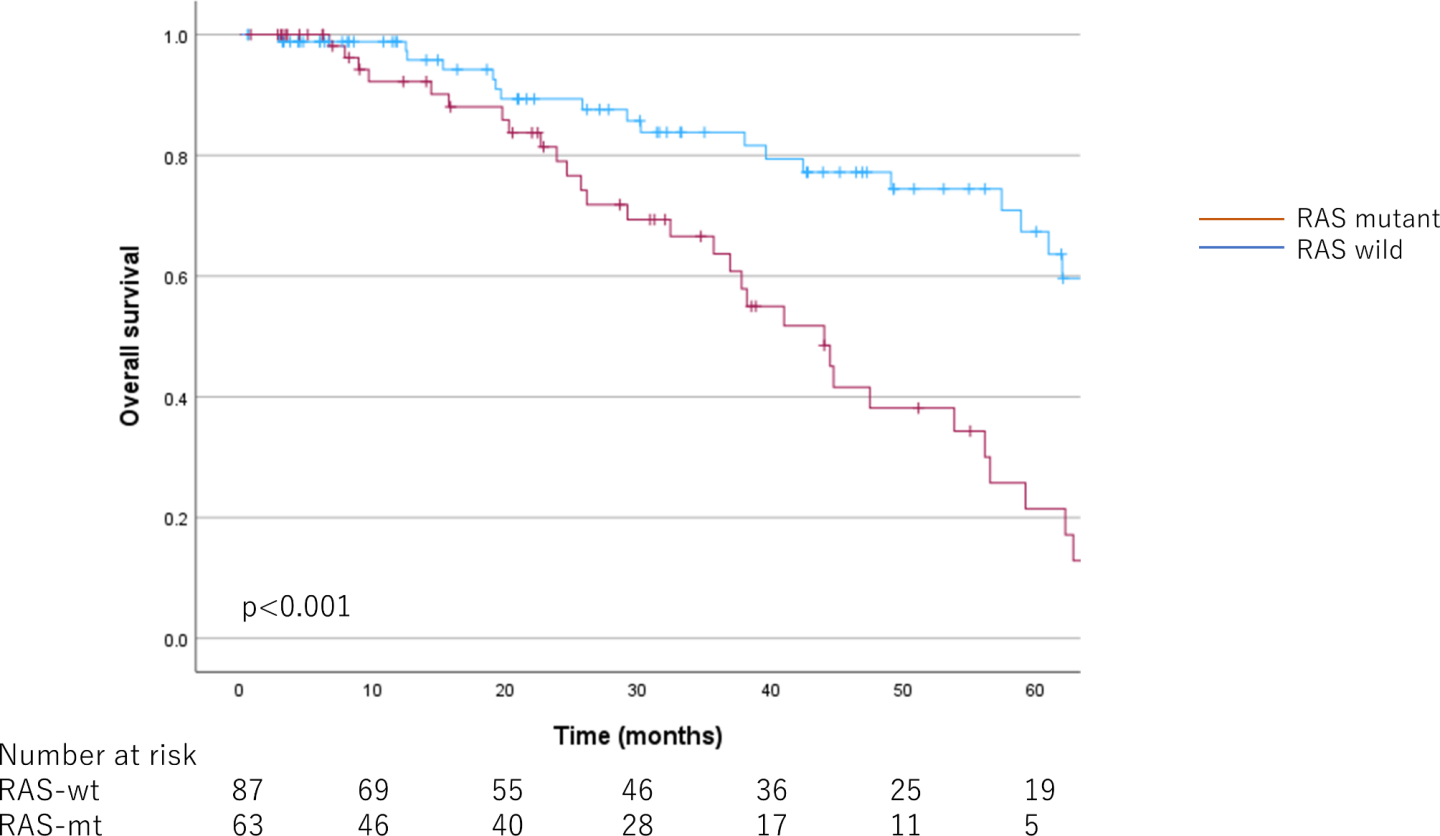
Kaplan-Meier curves by RAS status for overall survival

**Fig. 2:**
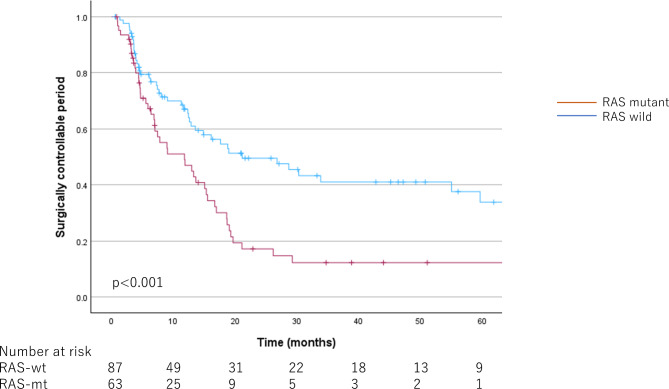
Kaplan-Meier curves by RAS status for surgically controllable period

**Fig. 3:**
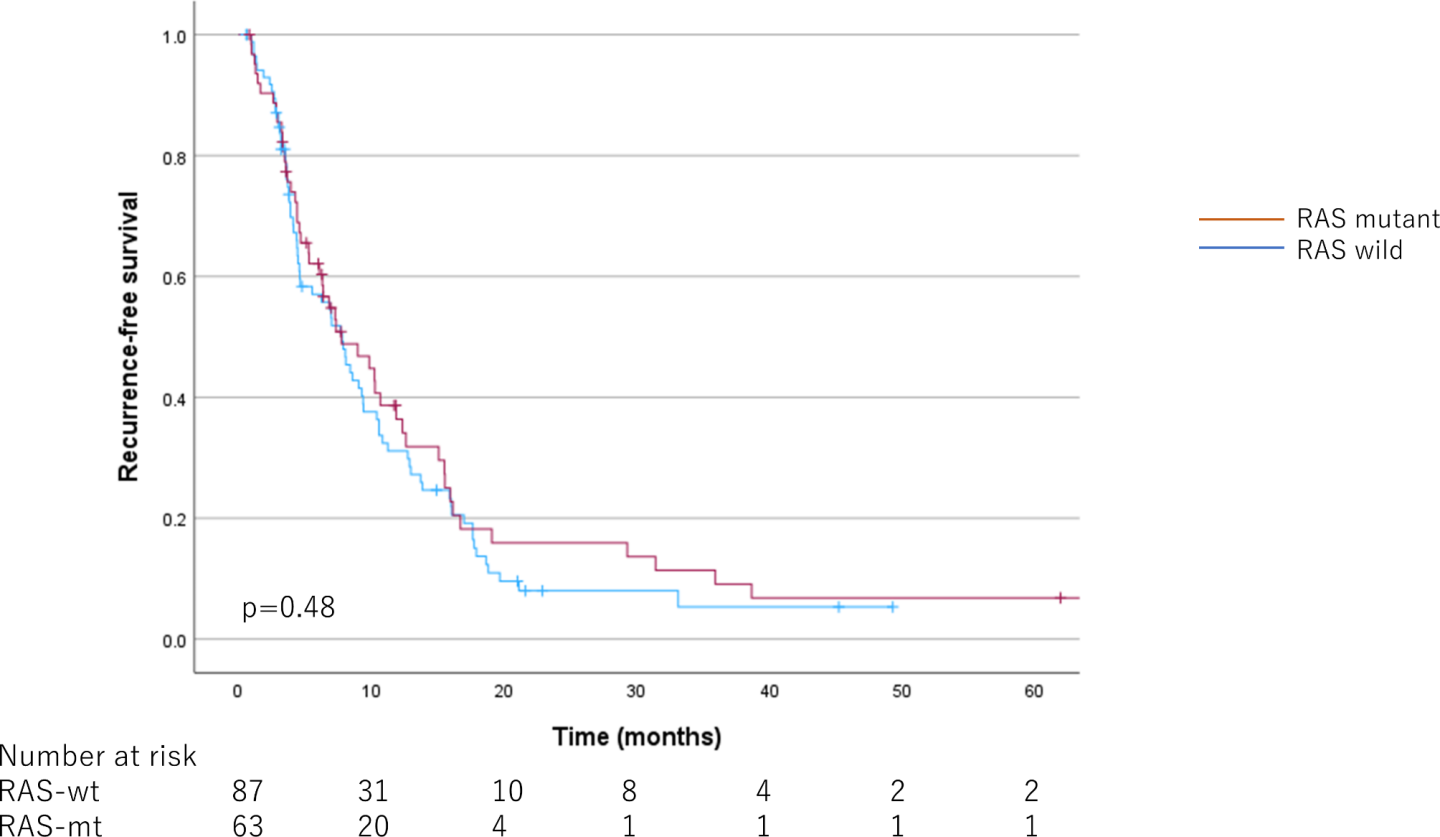
Kaplan-Meier curves by RAS status for recurrence-free survival

Median follow-up time for RAS-mt was 25.70 [20.53–34.73] months, and for RAS-wt was 31.40 [21.60–42.70] months. The survival curves for OS, SCP, and RFS according to RAS status are shown in Figs. 1 and 2, and 3, respectively. The OS and SCP were significantly shorter in RAS-mt. The median OS for RAS-mt was 44.03 [36.19–51.87] months, while for RAS-wt was 70.03 [58.00–82.07] months (*p* < 0.001). The median SCP was 11.80 [4.97–18.63] months for RAS-mt and 21.13 [8.19–34.07] months for RAS-wt (*p* < 0.001). However, no difference was observed in RFS by RAS status; median RFS was 7.00 [5.37–8.63] months for RAS-mt, while 8.03 [5.99–10.08] months for RAS-wt (*p* = 0.48).

In a subanalysis of KRAS codon 12 and 13 mutations, OS was shorter than RAS-wt for both codon 12 and codon 13 mutations. SCP was shorter for only codon 13 mutations. RFS was comparable to RAS -wt for both codon 12 and codon 13 mutations (Supplementary Figs. 1–6).

The recurrence pattern is shown in Supplementary Tables 2 and 3.

### Prognostic factors

In the analysis of prognostic factors for OS, RAS-mt (*p* < 0.001), right-sidedness (*p* = 0.07), size of metastasis > 5 cm (*p* = 0.02) and CA 19 − 9 > 100 U/mL (*p* = 0.08) were *p* < 0.1 in univariate analysis, and RAS-mt (HR: 3.37, 95% CI: 1.76–6.47, *p* < 0.001) and size of metastasis > 5 cm (HR: 2.48, 95% CI: 1.31–4.70, *p* = 0.005) were identified as independent poor prognostic factors in multivariate analysis (Table 2).

**Table 2:** Univariate and multivariate analysis for overall survival

Category	Variable	OS			
		Univariate		Multivariate	
		MST	P value	HR [95% CI]	P value
Age	> 60 y/o	60.93	0.14		
	≤ 60 y/o	56.53			
Sex	Male	57.40	0.85		
	Female	59.20			
RAS	Wild	70.03	< 0.001	Ref	
	Mutant	44.03		3.37 [1.76–6.47]	< 0.001*
Sidedness	Right	37.80	0.07	Ref	
	Left	60.93		0.97 [0.49–1.94]	0.93
T stage	T1–2	58.87	0.46		
	T3–4	57.40			
N stage	N0	57.40	0.35		
	N+	70.03			
Time to CRLM	Synchronous	56.13	0.22		
	Metachronous	62.20			
CRLM number	< 5	61.97	0.53		
	5≤	56.13			
CRLM size	≤ 5 cm	61.97	0.02	Ref	
	5 cm<	44.47		2.48 [1.31–4.70]	0.005*
Bilobar liver disease	Unilobar	56.53	0.41		
	Bilobar	60.93			
Prehepatic resection chemotherapy	Absent	56.53	0.51		
	Present	60.93			
Posthepatic resection chemotherapy	Absent	59.20	0.36		
	Present	53.83			
Pathological resection margin	-	58.87	0.39		
	+	39.63			
CEA	≤ 100ng/mL	59.20	0.70		
	> 100ng/mL	56.53			
CA 19 − 9	≤ 100U/mL	61.97	0.08	Ref	
	> 100U/mL	49.07		1.14 [0.62–2.09]	0.68

Abbreviations: OS, overall survival; MST, median survival time; HR, hazard ratio; CI, confidence interval; y/o, years old; CRLM, colorectal cancer liver metastasis; CEA, carcinoembryonic antigen; CA, carbohydrate antigen

*: *p* < 0.05

For SCP, RAS-mt (*p* < 0.001), size of metastasis > 5 cm (*p* = 0.06), and CA 19 − 9 > 100 U/mL (*p* = 0.053) were *p* < 0.1 in univariate analysis, and RAS-mt (HR: 2.20, 95% CI: 1.40–3.44, *p* < 0.001) and size of metastasis > 5 cm (HR: 1.75, 95% CI: 1.08–2.84, *p* = 0.02) were identified as independent poor prognostic factors in multivariate analysis (Table 3).

**Table 3:** Univariate and multivariate analysis for surgically controllable period

Category	Variable	SCP			
		Univariate		Multivariate	
		MST	p-value	HR [95% CI]	p-value
Age	> 60 y/o	13.57	0.94		
	≤ 60 y/o	17.63			
Sex	Male	16.13	0.69		
	Female	13.27			
RAS	Wild	21.13	< 0.001	Ref	
	Mutant	11.80		2.20 [1.40–3.44]	< 0.001*
Sidedness	Right	12.97	0.24		
	Left	17.63			
T stage	T1–2	29.27	0.39		
	T3–4	15.07			
N stage	N0	14.87	0.68		
	N+	15.33			
Time to CRLM	Synchronous	15.33	0.80		
	Metachronous	12.97			
CRLM number	< 5	14.87	0.49		
	5≤	15.33			
CRLM size	≤ 5 cm	17.63	0.06	Ref	
	5 cm<	12.43		1.75 [1.08–2.84]	0.02*
Bilobar liver disease	Unilobar	13.63	0.53		
	Bilobar	16.13			
Prehepatic resection chemotherapy	Absent	12.97	0.05		
	Present	18.70			
Posthepatic resection chemotherapy	Absent	15.07	0.63		
	Present	16.97			
Pathological resection margin	-	15.33	0.91		
	+	12.87			
CEA	Low	14.87	0.88		
	High	16.70			
CA 19 − 9	Low	18.63	0.053	Ref	
	High	11.87		1.21 [0.75–1.95]	0.43

Abbreviations: SCP, surgically controllable period; MST, median survival time; HR, hazard ratio; CI, confidence interval; y/o, years old; CRLM, colorectal cancer liver metastasis; CEA, carcinoembryonic antigen; CA, carbohydrate antigen

*: *p* < 0.05

For RFS, female sex (*p* = 0.09), pathological T3-4 (*p* = 0.06), synchronous metastasis (*p* = 0.06), and absence of preoperative chemotherapy (*p* = 0.054) were *p* < 0.1 in univariate analysis, and only preoperative chemotherapy was identified as an independent favorable prognostic factor in the multivariate analysis (HR: 0.67; 95% CI: 0.45–1.00; *p* = 0.049) (Supplementary Table 4).

### Risk factor and prognosis for short SCP

When the included patients were divided into short SCP and the other, only RAS-mt (*p* = 0.059) was *p* < 0.1 in univariate analysis. Multivariate analysis of RAS status and Beppu nomogram factors combined extracted RAS status as the only independent risk factor for short SCP (OR: 2.31, 95% CI: 1.08–4.95, *p* = 0.03) (Supplementary Table 5) [20]. The results of the multivariate analysis of all factors are shown in Supplementary Table 6.

**Fig. 4:**
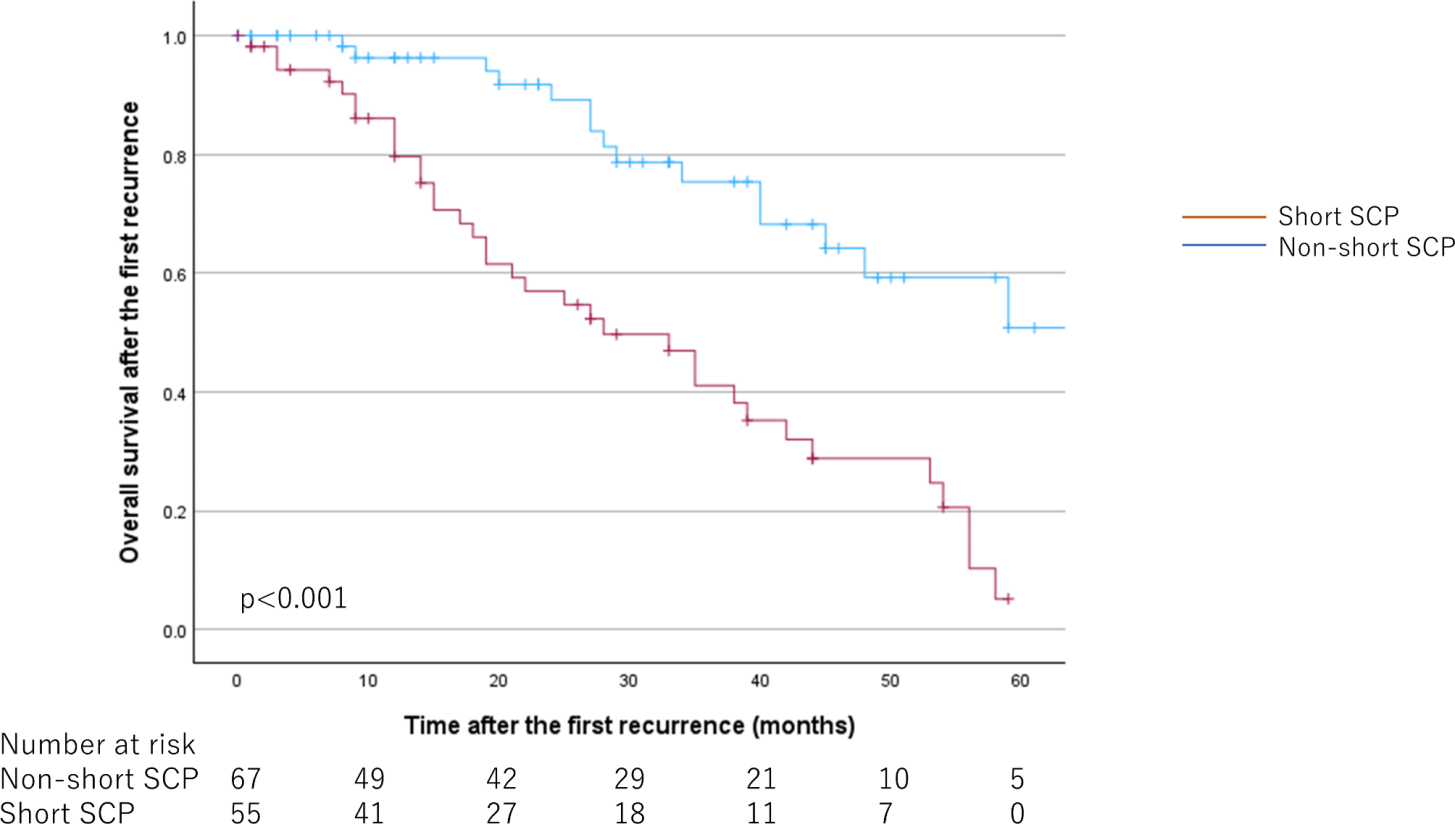
Kaplan-Meier curves by surgically controllable period for overall survival after the first recurrence

When comparing the long-term prognosis of patients with short SCP and non-short SCP, OS after the first recurrence was significantly worse in the short SCP group: the median OS after the first recurrence for short SCP was 28.00 [15.96–40.04] months, while for non-short SCP was 66.00 [43.04–88.07] months (*p* < 0.001). (Fig. 4).

## Discussion

In the present study, we found an association between RAS-mt and a shorter SCP, coupled with diminished OS after hepatectomy. Notably, SCP less than one year demonstrated a significant correlation with poor OS after the first recurrence of the initial hepatectomy.

A novel prognostic measure, SCP, was employed in this study, which is similar to the concept of “TSF” introduced by Oba et al. [21]. TSF was defined as the duration from the initial hepatectomy to the first recurrence with an unresectable status. The definition of SCP deviates from that of TSF, reflecting distinctions in our treatment approach for CRLM. Specifically, p-NAC is administered in our institution to patients with short disease-free intervals, such as recurrence within six months after curative liver resection, even when recurrence is considered resectable by repeat surgery. These patients are surgically favorable but oncologically unfavorable [23]. The disease-free interval is associated with a risk of recurrence, although not with OS [24]. If post-p-NAC imaging examination reveals no disease progression, additional surgical treatment is indicated. Conversely, if disease progression during chemotherapy renders the recurrence unresectable, the patient is categorized as having SF at the decision date. In multidisciplinary treatment, treatment is developed by many professions. The choice of chemotherapy drug and the explanation to the patient will differ depending on whether the patient is in the SCP or not. The concept “SCP” is useful because it allows multidisciplinary professionals to share whether a patient is in the SCP. Within a multidisciplinary treatment framework, we posit SCP as a more clinically relevant and pragmatic indicator in real-world scenarios.

Our results demonstrated that RAS-mt was an independent poor prognostic factor for both SCP and OS, as well as a risk factor for short SCP. Few previous reports have highlighted the association between RAS mutation and SCP. Wensink et al. reported that primary tumor side, stage, RAS/BRAF status, and the size and number of liver metastases were associated with early residual liver recurrence after local treatment [25]. Okuno et al. reported that RAS mutations were associated with unsalvageable and early recurrence after hepatic resection [26]. In this study, RAS-mt CRLM patients had a higher risk of developing SF in the short postoperative period, especially within the first year after hepatectomy, thereby increasing the risk of diminished OS. The shorter OS after the first recurrence in patients with short SCP suggests systemic recurrences.

In comparing survival curves between RAS-mt and RAS-wt patients, RAS-mt was worse in the SCP, but the RFS curve was similar. One possible reason is that current oncologic therapy with molecular-targeted agents of anti-EGFR and anti-VFGF drugs controlled the disease for a longer period of time in RAS-wt patients, whereas only anti-VEGF agents were available for RAS-mt patients. Another is that RAS-mt patients did not receive surgical intervention after recurrence, suggesting that RAS-mt is more likely to be a systemic disease, especially after recurrence.

In our series, patients with RAS-mt showed lung and liver recurrences more frequently (Supplementary Table 4). Lung recurrences were significantly more common in RAS-mt patients than in those with RAS-wt. Vauthey et al. noted that pulmonary recurrences were more prevalent in RAS-mt patients and that their prognosis after liver resection was notably poor [18]. Additionally, micrometastases likely contribute to liver recurrences in RAS-mt patients. Zhang et al. reported that patients with RAS-mt CRLM tended to have more micrometastases and narrower margins after liver resection [27]. Our findings suggest that RAS-mt patients also showed a trend towards more peritoneal recurrences, although this difference was not statistically significant.

The biological properties of RAS isoforms and codons are becoming better known. We performed a subanalysis of KRAS codon 12 and 13 mutations with respect to prognosis and relapse pattern. RAS codon 13 was particularly strongly associated with poor SCP and pulmonary recurrence. This result is consistent with Margonis et al. [28–30]. In recent years, the development of drugs targeting specific codons has been underway. Further individualization of treatment strategies, including the choice of modality, will be necessary, taking into account RAS isoforms and codons as well as the presence or absence of RAS mutations.

Given the poor prognosis post-hepatectomy and after initial recurrence, it’s crucial to ensure that liver surgeries in RAS-mt patients are curative and undertaken cautiously. On the other hand, patients who could benefit from resection for resectable lesions should not be overlooked. P-NAC has shown promise in eliminating micrometastases [31–34]. In hepatectomy in RAS mutation, careful indication selection by p-NAC may improve the prognosis after liver resection, and the possibility of short SCP needs to be noted. In current multidisciplinary treatment, the concept of SCP is expected to contribute to more efficient information sharing among providers and patients.

A limitation of this study is its retrospective nature because of the possibility of selection bias and missing information that may have influenced the results. The number of cases was limited because this was a single-center study. Patients with extrahepatic disease were excluded because SCP can be affected by extrahepatic disease. However, considering the limited number of reports on SCP and RAS, this study might help in the development of future treatments.

## Conclusion

In conclusion, the results of this study indicate that RAS mutations need to be considered for strict surgical indications with p-NAC and thorough preoperative examination, considering the possibility of short SCP.

## Electronic supplementary material

Below is the link to the electronic supplementary material.

Supplementary Material 1

Supplementary Material 2

Supplementary Material 3

Supplementary Material 4

Supplementary Material 5

Supplementary Material 6

Supplementary Material 7

Supplementary Material 8

## Data Availability

The data that support the findings of this study are not openly available due to reasons of sensitivity and are available from the corresponding author upon reasonable request. Data are located in controlled access data storage at National Cancer Center Hospital.
