# Supplementary figures and images for: RAS mutation associated with short surgically controllable period in colorectal liver metastases: a retrospective study
Source: World J Surg Oncol. 2024 Sep 12;22:247. doi: 10.1186/s12957-024-03529-9 (PMC11391794; doi:10.1186/s12957-024-03529-9)

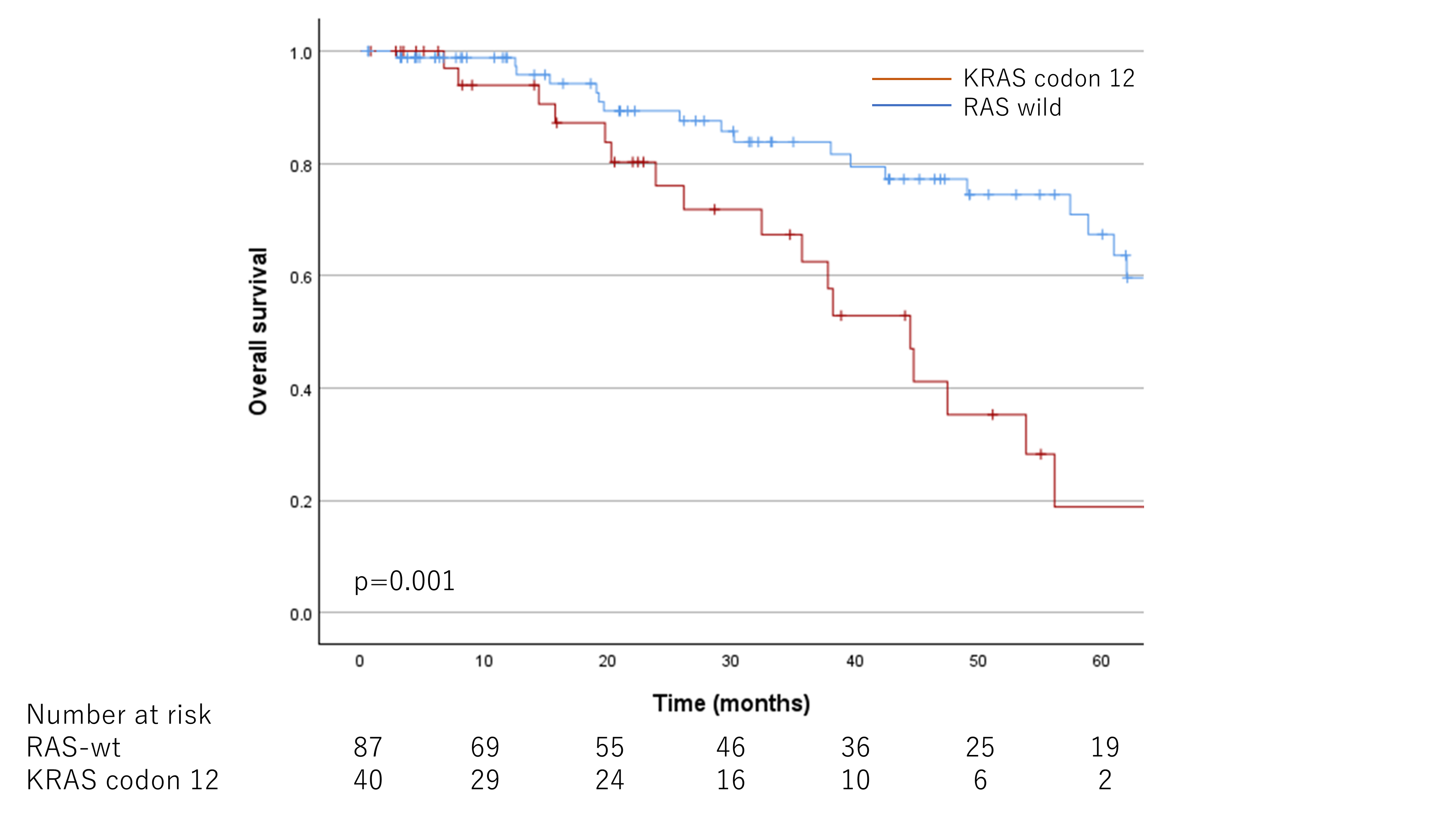

Supplement: Supplementary file 1 — Supplementary Material 1 [file 12957_2024_3529_MOESM1_ESM.tif]

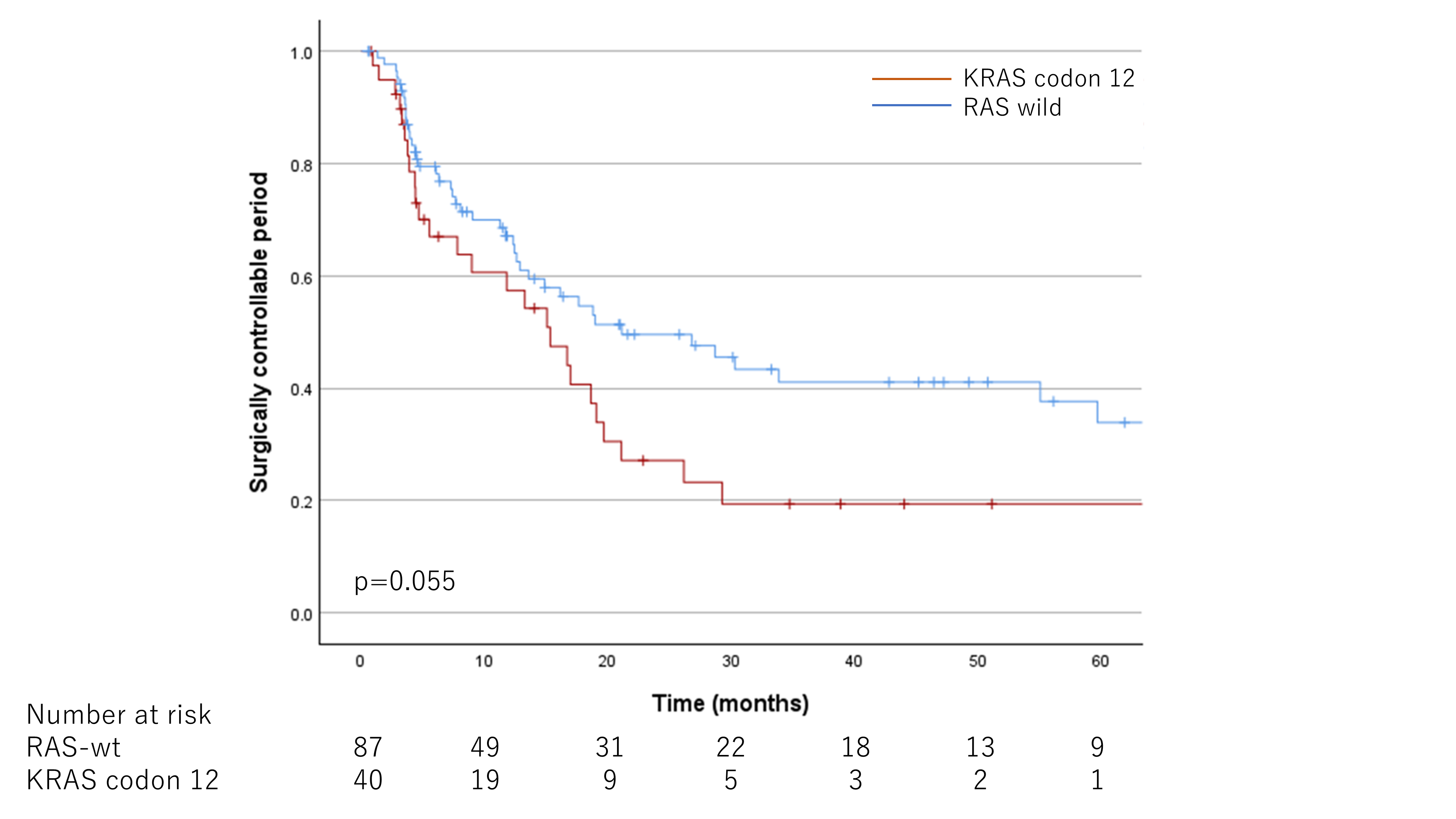

Supplement: Supplementary file 2 — Supplementary Material 2 [file 12957_2024_3529_MOESM2_ESM.tif]

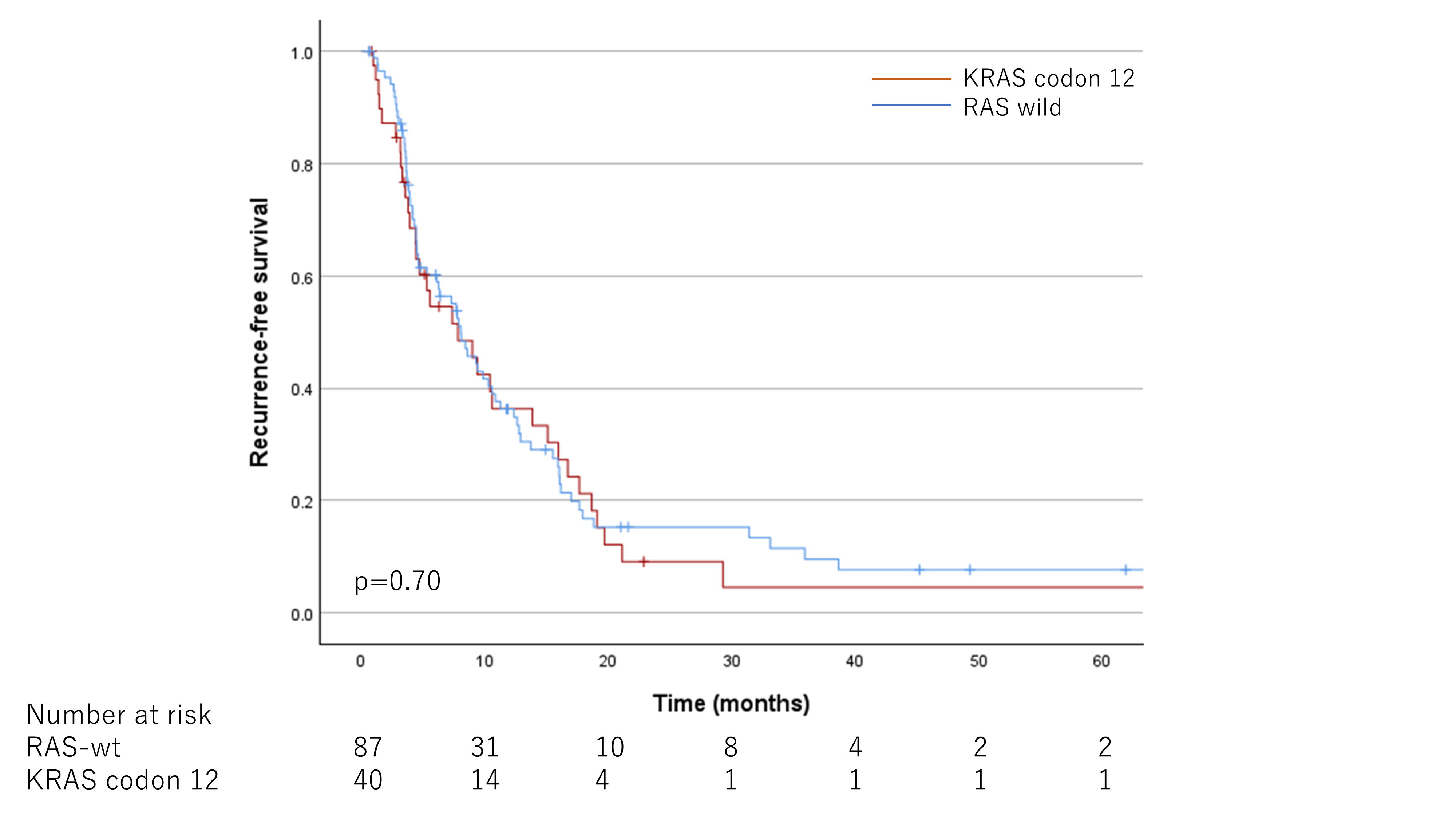

Supplement: Supplementary file 3 — Supplementary Material 3 [file 12957_2024_3529_MOESM3_ESM.tif]

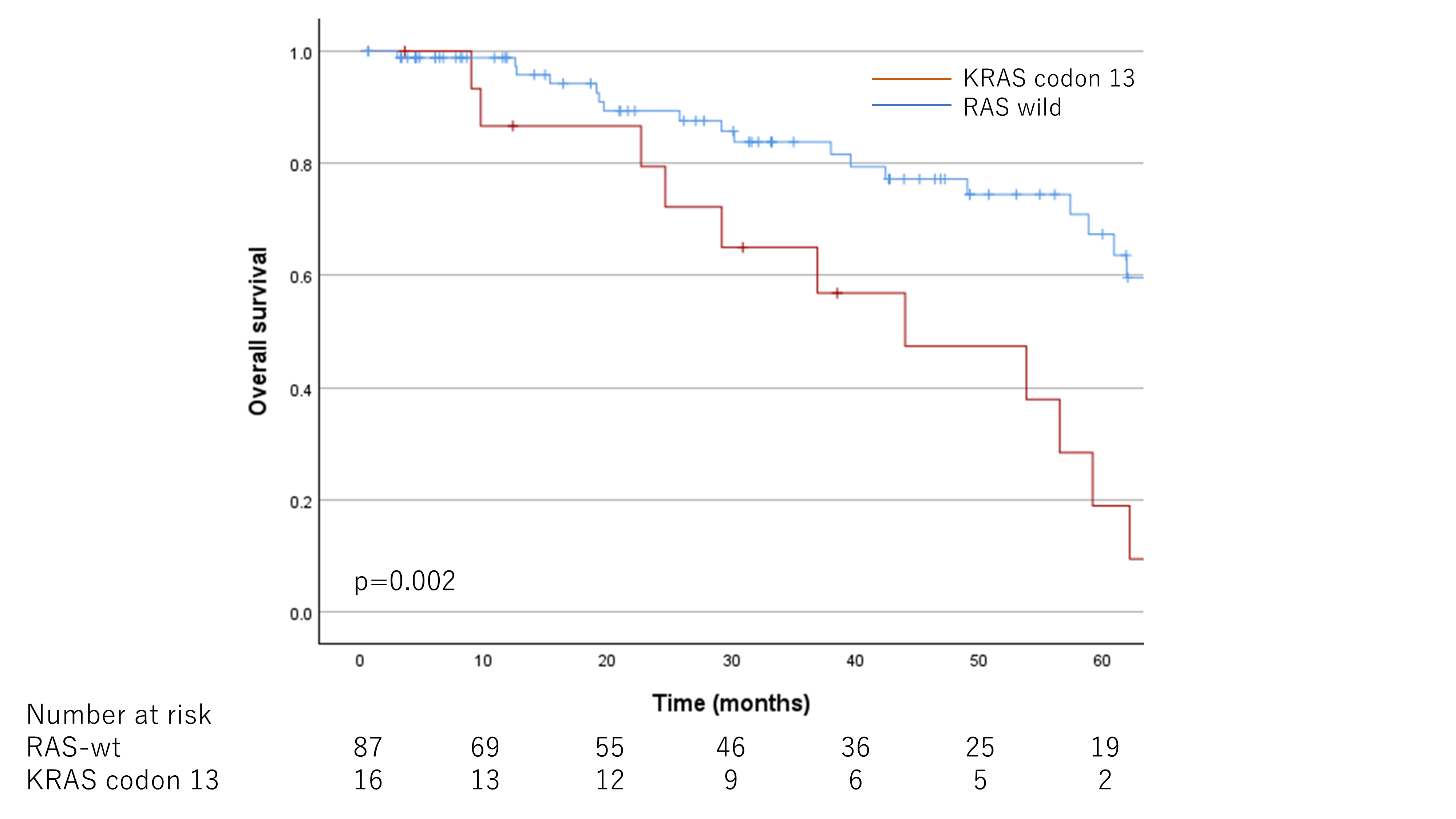

Supplement: Supplementary file 4 — Supplementary Material 4 [file 12957_2024_3529_MOESM4_ESM.tif]

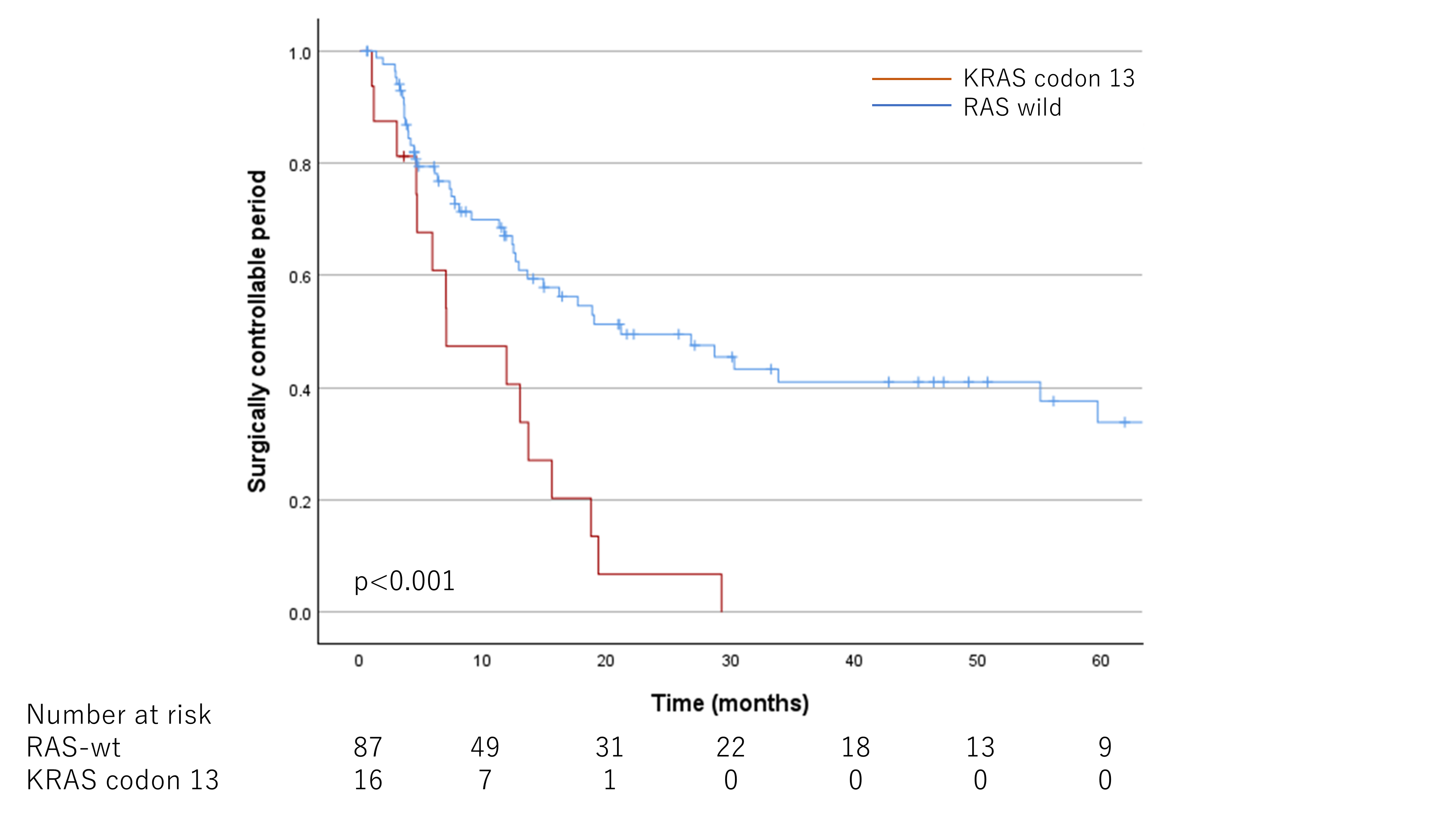

Supplement: Supplementary file 5 — Supplementary Material 5 [file 12957_2024_3529_MOESM5_ESM.tif]

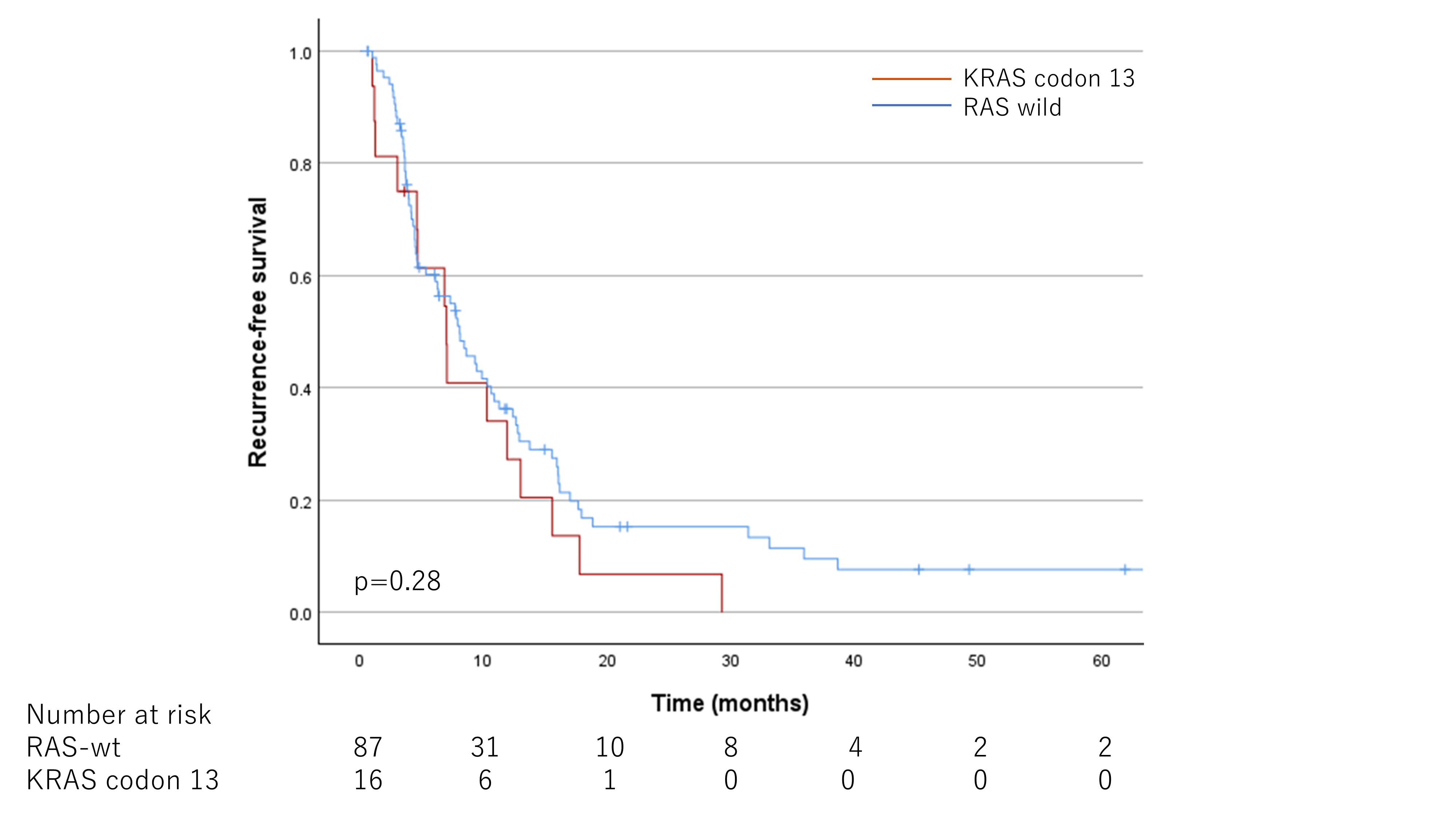

Supplement: Supplementary file 6 — Supplementary Material 6 [file 12957_2024_3529_MOESM6_ESM.tif]
