# Supplementary material for: RAS mutation associated with short surgically controllable period in colorectal liver metastases: a retrospective study
Source: World J Surg Oncol. 2024 Sep 12;22:247. doi: 10.1186/s12957-024-03529-9 (PMC11391794; doi:10.1186/s12957-024-03529-9)
Supplement: Supplementary file 7 — Supplementary Material 7 [file 12957_2024_3529_MOESM7_ESM.docx]

**Supplementary Table 1: Details of preoperative chemotherapy**

|  | Preoperative chemotherapy |
| --- | --- |
|  | N=57 |
| Chemotherapy |  |
| 5-FU, oxaliplatin | 45 (78.9%) |
| 5-FU, irrinotecan | 5 (8.8%) |
| 5-FU, oxaliplatin, irrinotecan | 1 (1.8%) |
| Only 5-FU | 4 (7.0%) |
| No administration | 0 (0%) |
| Unknown | 2 (3.5%) |
| Biologic target therapy |  |
| Panitumumab | 21 (36.8%) |
| Avastin | 18 (31.6%) |
| Cetuximab | 3 (5.3%) |
| No administration | 13 (22.8%) |
| Unknown | 2 (3.5%) |
| Number of cycles |  |
| <6 | 2 (3.5%) |
| ≥6 | 24 (42.1%) |
| Unknown | 31 (54.4%) |

Abbreviation: FU, fluorouracil.

**Supplementary Table 2: Recurrence site after hepatectomy**

|  |  | RAS status | |  |
| --- | --- | --- | --- | --- |
|  | Total | RAS-mt | RAS-wt | p-value |
| Number of recurrence organ |  |  |  | 0.47 |
| None | 27 (18.0) | 10 (15.9) | 17 (19.5) |  |
| Single | 83 (55.3) | 33 (52.4) | 50 (57.5) |  |
| Multiple | 40 (26.7) | 20 (31.7) | 20 (23.0) |  |
| Recurrence organ |  |  |  |  |
| Liver | 73 (48.7) | 25 (39.7) | 48 (55.2) | 0.06 |
| Lung | 45 (30.0) | 28 (44.4) | 17 (19.5) | 0.001* |
| Lymph node | 27 (18.0) | 14 (22.2) | 13 (14.9) | 0.25 |
| Peritoneum | 14 (9.3) | 9 (14.3) | 5 (5.7) | 0.08 |
| Adrenal | 4 (2.7) | 0 (0) | 4 (4.6) | 0.14 |
| Bone | 5 (3.3) | 1 (1.6) | 4 (4.6) | 0.40 |
| Local | 4 (2.7) | 1 (1.6) | 3 (3.4) | 0.64 |
| Ovary | 1 (0.7) | 0 (0) | 1 (1.1) | 1 |

Abbreviations: RAS-mt, RAS mutant type; RAS-wt, RAS wild type.

*: p<0.05

**Supplementary Table 3: Recurrence site after hepatectomy of KRAS codon 12 and 13 mutations**

|  |  | RAS-mt | | | |
| --- | --- | --- | --- | --- | --- |
|  | RAS-wt | Codon 12 | p-value | Codon 13 | p-value |
| Number of recurrence organ |  |  | 0.36 |  | 0.52 |
| None | 17 (19.5) | 7 (17.5) |  | 1 (6.3) |  |
| Single | 50 (57.5) | 19 (47.5) |  | 11 (68.8) |  |
| Multiple | 20 (23.0) | 14 (35.0) |  | 4 (25.0) |  |
| Recurrence organ |  |  |  |  |  |
| Liver | 48 (55.2) | 17 (42.5) | 0.18 | 5 (31.3) | 0.08 |
| Lung | 17 (19.5) | 18 (45.0) | 0.003* | 7 (43.8) | 0.052 |
| Lymph node | 13 (14.9) | 9 (22.5) | 0.30 | 6 (37.5) | 0.07 |
| Peritoneum | 5 (5.7) | 5 (12.5) | 0.29 | 3 (18.8) | 0.11 |
| Adrenal | 4 (4.6) | 0 (0) | 0.31 | 0 (0) | 1.00 |
| Bone | 4 (4.6) | 0 (0) | 0.31 | 0 (0) | 1.00 |
| Local | 3 (3.4) | 0 (0) | 0.55 | 1 (6.3) | 0.50 |
| Ovary | 1 (1.1) | 0 (0) | 1.00 | 0 (0) | 1.00 |

Abbreviations: RAS-mt, RAS mutant type; RAS-wt, RAS wild type.

*: p<0.05

**Supplementary Table 4: Univariate and multivariate analysis for recurrence-free survival**

| Category | Variable | RFS | | | |
| --- | --- | --- | --- | --- | --- |
|  |  | Univariate | | Multivariate | |
|  |  | MST | p-value | HR [95% CI] | p-value |
| Age | >60 y/o | 8.40 | 0.14 |  |  |
|  | ≤60 y/o | 7.33 |  |  |  |
| Sex | Male | 8.40 | 0.09 | 0.88 [0.60 - 1.29] | 0.51 |
|  | Female | 6.87 |  | Ref |  |
| RAS | Wild | 8.03 | 0.35 |  |  |
|  | Mutant | 7.00 |  |  |  |
| Sidedness | Right | 5.53 | 0.37 |  |  |
|  | Left | 8.03 |  |  |  |
| T stage | T1 - 2 | 11.23 | 0.06 | Ref |  |
|  | T3 - 4 | 7.33 |  | 1.54 [0.81 - 2.94] | 0.19 |
| N stage | N0 | 9.03 | 0.14 |  |  |
|  | N+ | 6.80 |  |  |  |
| Time to CRLM | Synchronous | 6.80 | 0.06 | Ref |  |
|  | Metachronous | 9.03 |  | 0.71 [0.47 - 1.05] | 0.09 |
| CRLM number | <5 | 8.07 | 0.23 |  |  |
|  | 5≤ | 4.63 |  |  |  |
| CRLM size | ≤5cm | 8.40 | 0.19 |  |  |
|  | 5cm< | 6.23 |  |  |  |
| Bilobar liver disease | Unilobar | 8.03 | 0.15 |  |  |
|  | Bilobar | 6.07 |  |  |  |
| Prehepatic resection chemotherapy | Absent | 6.97 | 0.054 | Ref |  |
|  | Present | 9.37 |  | 0.67 [0.45 - 1.00] | 0.049* |
| Posthepatic resection chemotherapy | Absent | 7.80 | 0.42 |  |  |
|  | Present | 7.67 |  |  |  |
| Pathological resection margin | - | 7.80 | 0.22 |  |  |
|  | + | 6.30 |  |  |  |
| CEA | Low | 7.73 | 0.21 |  |  |
|  | High | 11.87 |  |  |  |
| CA 19 - 9 | Low | 8.03 | 0.15 |  |  |
|  | High | 4.63 |  |  |  |

Abbreviations: RFS, recurrence-free survival; MST, median survival time; HR, hazard ratio; CI, confidence interval; y/o, years old; CRLM, colorectal cancer liver metastasis; CEA, carcinoembryonic antigen; CA, carbohydrate antigen.

*: p<0.05

**Supplementary Table 5: Univariate and multivariate risk analysis for the short surgically controllable period**

| Category | Variable | Univariate | | | | | Multivariate | |
| --- | --- | --- | --- | --- | --- | --- | --- | --- |
|  |  | SCP | | | |  |  |  |
|  |  | ≥1y | | <1y | | p-value | OR [95% CI] | p-value |
| Age | >60 y/o | 42 | 44% | 27 | 49% | 0.61 |  |  |
|  | ≤60 y/o | 53 | 56% | 28 | 51% |  |  |  |
| Sex | Male | 58 | 61% | 32 | 58% | 0.73 |  |  |
|  | Female | 37 | 39% | 23 | 42% |  |  |  |
| RAS | Wild | 61 | 64% | 26 | 47% | 0.059 | Ref |  |
|  | Mutant | 34 | 36% | 29 | 53% |  | 2.31 [1.08 - 4.95] | 0.03* |
| Sidedness | Right | 19 | 20% | 15 | 27% | 0.32 |  |  |
|  | Left | 76 | 80% | 40 | 73% |  |  |  |
| T stage | T1 - 2 | 13 | 14% | 5 | 9% | 0.45 |  |  |
|  | T3 - 4 | 82 | 86% | 50 | 91% |  |  |  |
| N stage | N0 | 38 | 40% | 20 | 36% | 0.73 | Ref |  |
|  | N+ | 57 | 60% | 35 | 64% |  | 0.90 [0.43 - 1.92] | 0.79 |
| Time to CRLM | Synchronous | 61 | 64% | 37 | 67% | 0.73 | Ref |  |
|  | Metachronous | 34 | 36% | 18 | 33% |  | 0.77 [0.35 - 1.73] | 0.53 |
| CRLM number | <5 | 70 | 74% | 40 | 73% | 1.00 | Ref |  |
|  | 5≤ | 25 | 26% | 15 | 27% |  | 0.85 [0.37 - 1.93] | 0.70 |
| CRLM size | ≤5cm | 77 | 81% | 39 | 71% | 0.16 | Ref |  |
|  | 5cm< | 18 | 19% | 16 | 29% |  | 2.30 [0.97 - 5.45] | 0.06 |
| Bilobar liver disease | Unilobar | 47 | 50% | 26 | 47% | 0.87 |  |  |
|  | Bilobar | 48 | 50% | 29 | 53% |  |  |  |
| Prehepatic resection chemotherapy | Absent | 55 | 58% | 38 | 69% | 0.22 |  |  |
|  | Present | 40 | 42% | 17 | 31% |  |  |  |
| Posthepatic resection chemotherapy | Absent | 81 | 85% | 45 | 82% | 0.65 |  |  |
|  | Present | 14 | 15% | 10 | 18% |  |  |  |
| Pathological resection margin | - | 74 | 78% | 40 | 73% | 0.55 |  |  |
|  | + | 21 | 22% | 15 | 27% |  |  |  |
| CEA | Low | 79 | 83% | 49 | 89% | 0.47 |  |  |
|  | High | 16 | 17% | 6 | 11% |  |  |  |
| CA 19 - 9 | Low | 70 | 77% | 35 | 67% | 0.24 | Ref |  |
|  | High | 21 | 23% | 17 | 33% |  | 1.19 [0.52 - 2.70] | 0.68 |

Abbreviations: SCP, surgically controllable period; OR, odds ratio; CI, confidence interval; y/o, years old; CRLM, colorectal cancer liver metastasis; CEA, carcinoembryonic antigen; CA, carbohydrate antigen.

*: p<0.05

**Supplementary Table 6: Multivariate risk analysis for the short surgically controllable period considering all factors**

| Category | Variable | Multivariate | |
| --- | --- | --- | --- |
|  |  | OR [95% CI] | p-value |
| Age | >60 y/o | 0.70 [0.31 – 1.56] | 0.38 |
|  | ≤60 y/o | Ref |  |
| Sex | Male | 1.23 [0.54 -2.78] | 0.63 |
|  | Female | Ref |  |
| RAS | Wild | Ref |  |
|  | Mutant | 2.55 [1.06 -6.15] | 0.04* |
| Sidedness | Right | Ref |  |
|  | Left | 0.76 [0.30 – 1.95] | 0.57 |
| T stage | T1 - 2 | Ref |  |
|  | T3 - 4 | 0.79 [0.21 -2.91] | 0.72 |
| N stage | N0 | Ref |  |
|  | N+ | 1.02 [0.45 -2.28] | 0.97 |
| Time to CRLM | Synchronous | Ref |  |
|  | Metachronous | 0.72 [0.30 -1.72] | 0.46 |
| CRLM number | <5 | Ref |  |
|  | 5≤ | 0.53 [0.18 -1.57] | 0.25 |
| CRLM size | ≤5cm | Ref |  |
|  | 5cm< | 3.15 [1.23 -8.09] | 0.02* |
| Bilobar liver disease | Unilobar | Ref |  |
|  | Bilobar | 1.58 [0.65 -3.87] | 0.32 |
| Prehepatic resection chemotherapy | Absent | Ref |  |
|  | Present | 0.71 [0.31 – 1.64] | 0.43 |
| Posthepatic resection chemotherapy | Absent | Ref |  |
|  | Present | 1.35 [0.49 -3.75] | 0.56 |
| Pathological resection margin | - | Ref |  |
|  | + | 1.61 [0.62 - 4.14] | 0.33 |
| CEA | Low | Ref |  |
|  | High | 0.20 [0.06 -0.74] | 0.02* |
| CA 19 - 9 | Low | Ref |  |
|  | High | 1.81 [0.70 – 4.65] | 0.22 |

Abbreviations: SCP, surgically controllable period; OR, odds ratio; CI, confidence interval; y/o, years old; CRLM, colorectal cancer liver metastasis; CEA, carcinoembryonic antigen; CA, carbohydrate antigen.

*: p<0.05
