# Supplementary material for: RAS mutation associated with short surgically controllable period in colorectal liver metastases: a retrospective study
Source: World J Surg Oncol. 2024 Sep 12;22:247. doi: 10.1186/s12957-024-03529-9 (PMC11391794; doi:10.1186/s12957-024-03529-9)
Supplement: Supplementary file 8 — Supplementary Material 8 [file 12957_2024_3529_MOESM8_ESM.docx]

**Supplementary Figure 1:Kaplan-Meier curve of KRAS codon 12 mutation for overall survival**

**Supplementary Figure 2: Kaplan-Meier curve of KRAS codon 12 mutation for surgically controllable period**

**Supplementary Figure 3: Kaplan-Meier curve of KRAS codon 12 mutation for recurrence-free survival**

**Supplementary Figure 4: Kaplan-Meier curve of KRAS codon 13 mutation for overall survival**

**Supplementary Figure 5: Kaplan-Meier curve of KRAS codon 13 mutation for surgically controllable period**

**Supplementary Figure 6: Kaplan-Meier curve of KRAS codon 13 mutation recurrence-free survival**
